# Osteoblast-targeted delivery of miR-33-5p attenuates osteopenia development induced by mechanical unloading in mice

**DOI:** 10.1038/s41419-017-0210-5

**Published:** 2018-02-07

**Authors:** Han Wang, Zebing Hu, Fei Shi, Jingjing Dong, Lei Dang, Yixuan Wang, Zhongyang Sun, Hua Zhou, Shu Zhang, Xinsheng Cao, Ge Zhang

**Affiliations:** 1 Department of Orthopedics, Affiliated Hospital of Air Force Aviation Medicine Research Institute, The Fourth Military Medical University, 100089 Beijing, China; 2 The Key Laboratory of Aerospace Medicine, Ministry of Education, The Fourth Military Medical University, 710032 Xi’an, Shaanxi China; 3 Institute for Advancing Translational Medicine in Bone & Joint Diseases, School of Chinese Medicine, Hong Kong Baptist University, Hong Kong SAR, China; 4 Department of Orthopedics, No. 454 Hospital of PLA, 210002 Nanjing, Jiangsu China

## Abstract

A growing body of evidence has revealed that microRNAs (miRNAs) play crucial roles in regulating osteoblasts and bone metabolism. However, the effects of miRNAs in osteoblast mechanotransduction remain to be defined. In this study, we investigated the regulatory effect of miR-33-5p in osteoblasts and tested its anti-osteopenia effect when delivered by an osteoblast-targeting delivery system in vivo. First, we demonstrated that miR-33-5p could promote the activity and mineralization of osteoblasts without influencing their proliferation in vitro. Then our data showed that supplementing miR-33-5p in osteoblasts by a targeted delivery system partially recovered the osteopenia induced by mechanical unloading at the biochemical, microstructural, and biomechanical levels. In summary, our findings demonstrate that miR-33-5p is a key factor in the occurrence and development of the osteopenia induced by mechanical unloading. In addition, targeted delivery of the mimics of miR-33-5p is a promising new strategy for the treatment of pathological osteopenia.

## Introduction

Mechanical load is a master regulator of bone formation and resorption, and bone tissue responds to the stimulus of mechanical load for growth or maintenance^1,2^. Bone loss due to mechanical unloading is characterized by an uncoupling of bone turnover: bone formation decreases while bone resorption increases^3,4^. This process mainly occurs in patients who require prolonged immobilization or bed rest and astronauts in a microgravity environment. With the aging of the population becoming more and more serious, the number of long-term bedridden patients is increasing. Microgravity is a special and comparatively thorough environment for mechanical unloading, which could cause rapid and considerable bone loss^5–7^. Many researchers have indicated that suppression of bone formation and activation of bone resorption are the main reasons for osteopenia induced by mechanical unloading^8,9^, in which the inhibition of bone formation is caused by the weakening of osteoblast activity. Thus the mechanism of how osteoblast activity is inhibited by mechanical unloading merits further research.

MicroRNAs (miRNAs) are a class of single-stranded noncoding RNA of approximately 22 nucleotides or less^10,11^. Normally, miRNAs are highly conserved in many species and could take part in the regulation of broad-spectrum biological processes by negatively regulating the translation of their target mRNAs^12^. In fact, some miRNAs have been shown to induce enhancing or weakening of osteoblast function under different mechanical stimulations. miR-3077-5p, -3090-5p, -3103-5p, -466i-3p, and -466h-3p, which correlate with the key genes of osteoblast differentiation as revealed by bioinformatics analysis, were dramatically different under cyclic mechanical strain in MC3T3-E1 cells^13,14^. In addition, some miRNAs are involved in the process by which fluid shear stress induces MC3T3-E1 cell differentiation, such as miR-20a, miR-21, miR-19b, miR-34a, miR-34c, miR-140, and miR-200b^15,16^. Similarly, miRNAs also play an important role in the mechanical unloading-induced reduction in osteoblast function. Wang et al. first revealed that miR-214 inhibits osteoblast function in the hindlimb unloading (HU) model, which simulated the bone loss induced by microgravity^17^. In addition, our previous work indicated that simulated microgravity upregulates the expression of miR-103, resulting in downregulation of Cav1.2 expression, inhibition of LTCC function, and inhibition of osteoblast proliferation^18,19^. Our findings also demonstrated that miR-132-3p participated in the regulation of bone loss induced by simulated microgravity and it can inhibit osteoblast differentiation by reducing Ep300 protein expression, which in turn resulted in suppression of the activity and acetylation of Runx2^20^. More interestingly, miR-33-5p was found to be sensitive to multiple mechanical environments. Our results showed that miR-33-5p could promote osteoblast differentiation by blocking the translation of its target gene Hmga2 and that microgravity or fluid shear stress influences osteoblast differentiation partially via miR-33-5p in MC3T3-E1 cells. Furthermore, we found that supplementation with miR-33-5p partially attenuated the inhibition of osteoblast differentiation by simulated microgravity in vitro^21^. Based on the above results, we aimed to verify whether miR-33-5p could influence the other functions of osteoblasts, and we further investigated the regulatory effect of miR-33-5p on bone formation in vivo.

As more miRNAs have been found to play key roles in many pathological processes, the value of miRNAs acting as therapeutic targets in many diseases has received more attention^22–24^. However, there are some challenges with the application of miRNA modulators in vivo^25^. First, the side effects of miRNA modulators in other tissues and organs could decrease the safety of miRNA modulators. Second, systemic application of miRNA modulators in vivo requires large doses, increasing the experimental cost. Third, the effective reaction time of miRNA modulators in vivo is not long enough, although chemical modification enhances their stability and slows their degradation^26–29^. To solve these problems, targeted delivery systems for miRNA modulators have been invented and upgraded continuously. For example, miR-122 was the first miRNA therapeutic target for disease. Inhibition of miR-122 by the method of locked nucleic acid was functional in the treatment of hepatitis C, and a phase II clinical trial was begun in 2012^30^. In addition, a miR-122 mimic delivered by the cationic lipid nanoparticle LNP-DP1 suppressed tumor growth and angiogenesis in hepatocellular carcinoma^31^. A TEPA-PCL polycation liposome delivery system was used to deliver miR-92a into the angiogenic endothelial cells to inhibit tumor angiogenesis^32^. To date, only a few bone tissue-specific miRNA delivery systems have been invented and preliminarily applied in bone research^33^. Among them, the (AspSerSer)_6_-liposome delivery system has been verified to specifically deliver miRNA modulators into osteoblasts in vivo where they regulate osteoblast function effectively and efficiently^34^.

In this study, a mimic or an inhibitor of miR-33-5p was used in MC3T3-E1 cells to investigate its gain- or loss-of-function in osteoblast activity in vitro. Moreover, a murine HU model was generated in vivo to further test the potential anti-osteoporosis effects of miR-33-5p targeting delivered by the (AspSerSer)_6_-liposome delivery system. To the best of our knowledge, this is the first report demonstrating that miR-33-5p exhibits protective effects against mechanical unloading-induced osteopenia in vivo.

## Results

### miR-33-5 promotes osteoblast activity in vitro

**Fig. 1:**
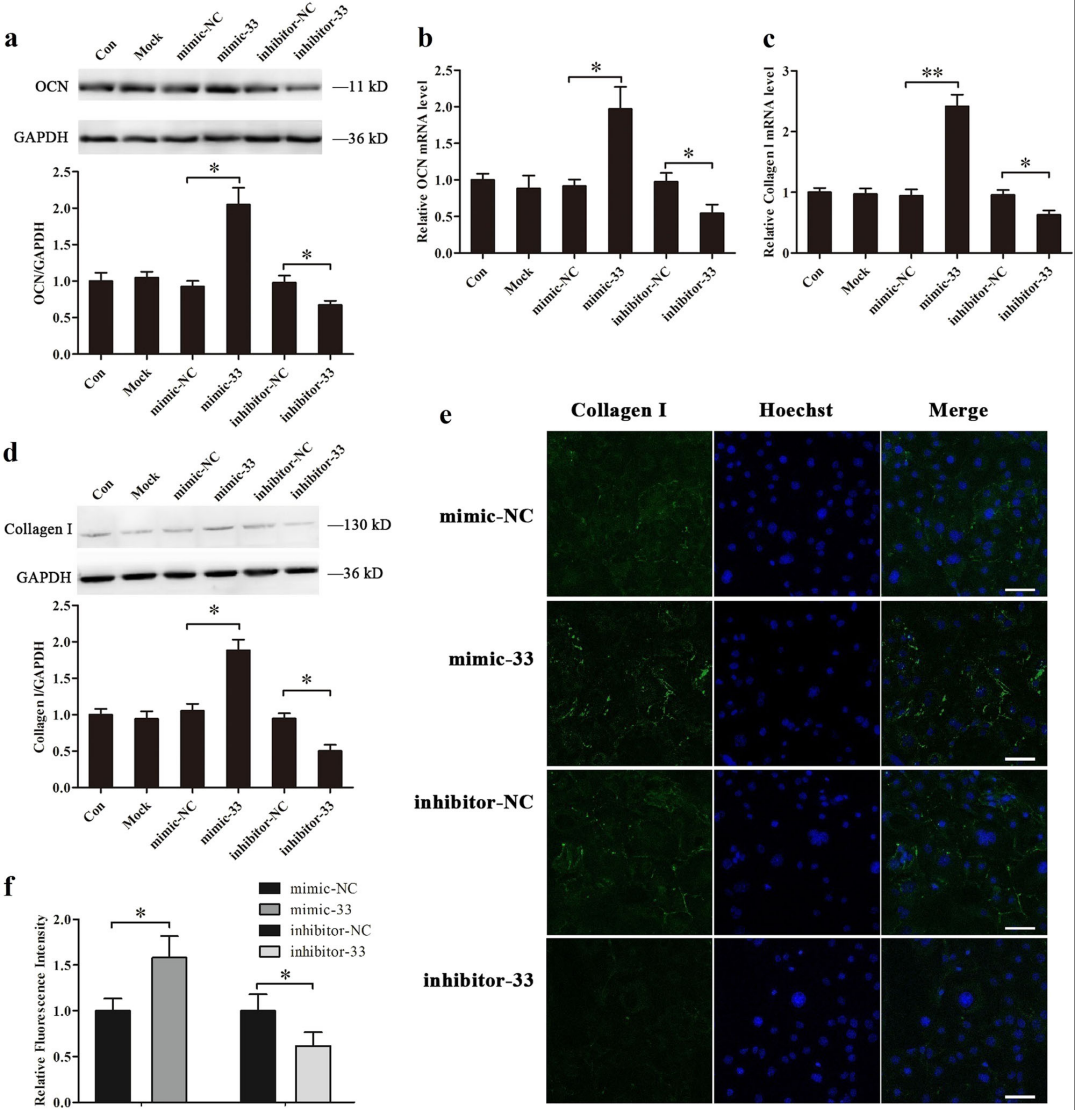
miR-33-5p promotes the osteoblast activity of MC3T3-E1 cells. **a**, **d** Western blot analysis of the changes in OCN and Collagen I protein levels in MC3T3-E1 cells after treatment with mimic-33, inhibitor-33, or their negative controls for 48 h. **b**, **c** qRT-PCR analysis of the changes in the mRNA expression levels of OCN and Collagen I in MC3T3-E1 cells after treatment with mimic-33, inhibitor-33, or their negative controls for 48 h. The values are shown relative to that of the control. **e** Immunostaining analysis of the changes in Collagen I expression after treatment with mimic-33, inhibitor-33, or their negative controls for 48 h. Green: Collagen I, blue: Hoechst staining of nuclei. All photomicrographs were recorded under identical exposure and magnification conditions. Scale bar, 20 µm. **f** The relative fluorescent intensity of Collagen I after treatment with mimic-33, inhibitor-33, or their negative controls for 48 h. The values of mimic-33-negative control or inhibitor-33-negative control are expressed as 1 arbitrary unit. The data are expressed as the mean ± SD of three replicates each. *P < 0.05, **P < 0.01 vs. the negative control

To investigate the effect of miR-33-5p on regulating osteoblast activity, we treated MC3T3-E1 cells with either mimic-33 or inhibitor-33. Intracellular miR-33-5p levels were significantly upregulated by mimic-33 treatment and markedly downregulated by inhibitor-33 treatment (Supplementary Fig. 1). Overexpression of miR-33-5p promoted mRNA and intracellular protein levels of osteocalcin (OCN) and collagen I, whereas knockdown of miR-33-5p inhibited the mRNA and intracellular protein expression of OCN (Fig. 1a, b) and collagen I (Fig. 1c, d). In addition, the collagen I proteins were further observed by immunofluorescence, and it was shown that the fluorescence intensity of collagen I proteins were upregulated by mimic-33 and downregulated by inhibitor-33 compared to the negative controls (Fig. 1e). And their differences were significant evaluated by semiquantitative analysis (Fig. 1f).

### The effect of miR-33-5p on osteoblast proliferation and mineralization

**Fig. 2:**
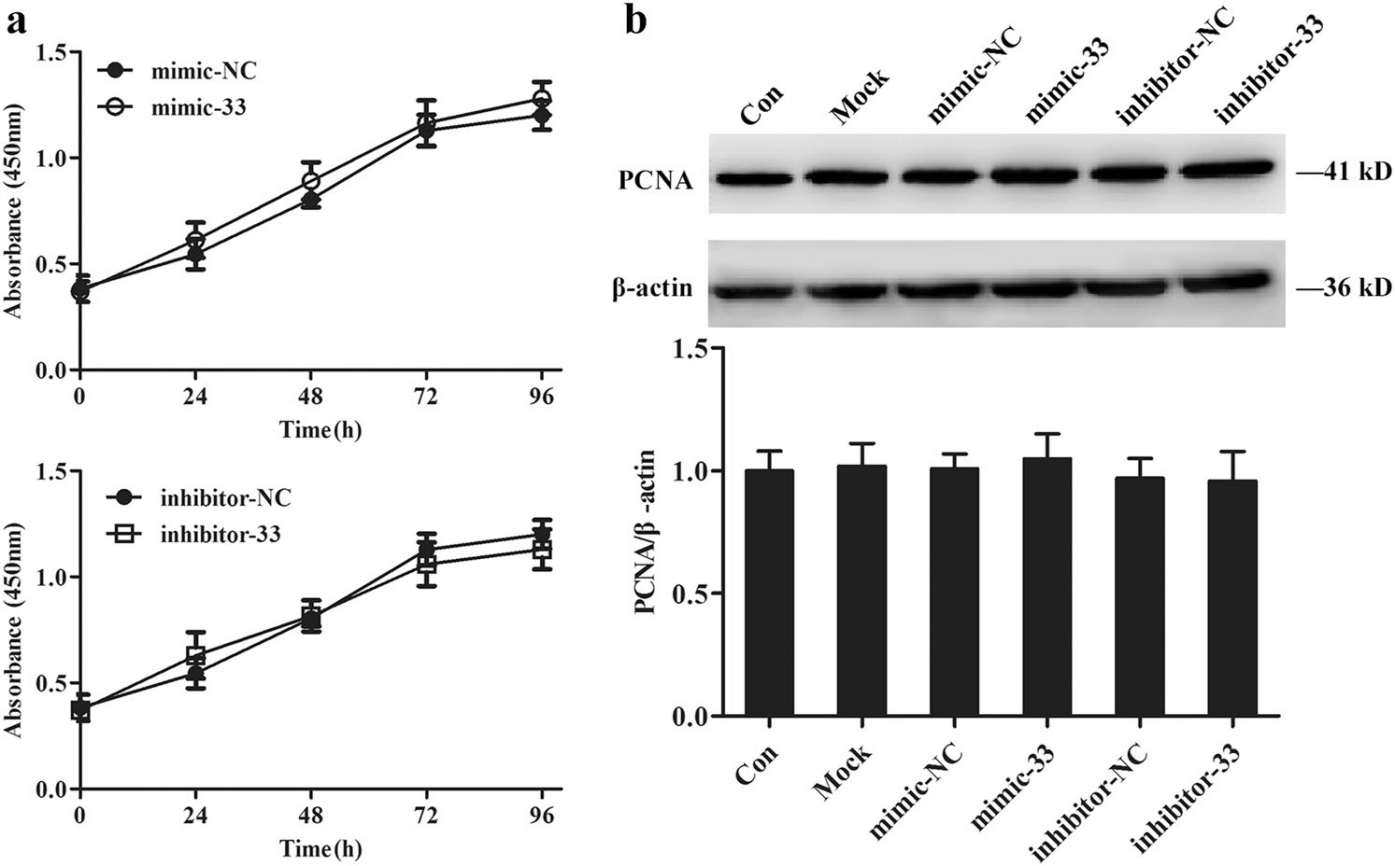
miR-33-5p nonsignificantly affects the proliferation of MC3T3-E1 cells. **a** WST-8 assay of the change in cell growth after treatment with mimic-33, inhibitor-33, or their negative controls. **b** Western blot analysis of the changes in PCNA protein levels in MC3T3-E1 cells after treatment with mimic-33, inhibitor-33, or their negative controls for 48 h. The data are expressed as the mean ± SD of three replicates each

After treating the MC3T3-E1 cells with mimic-33 or inhibitor-33, we further tested cellular proliferation and mineralization. After a 48-h transfection with miR-33-5p mimic/inhibitor or with the negative controls, no significant change was found between the growth curves as measured by the WST-8 assay (Fig. 2a). To further confirm the results of the WST-8 assay, we tested the expression of proliferating cell nuclear antigen (PCNA) proteins as a marker of proliferation. Consistently, the PCNA protein levels were non-significantly changed after treatment with mimic-33, inhibitor-33, or their negative controls (Fig. 2b).

**Fig. 3:**
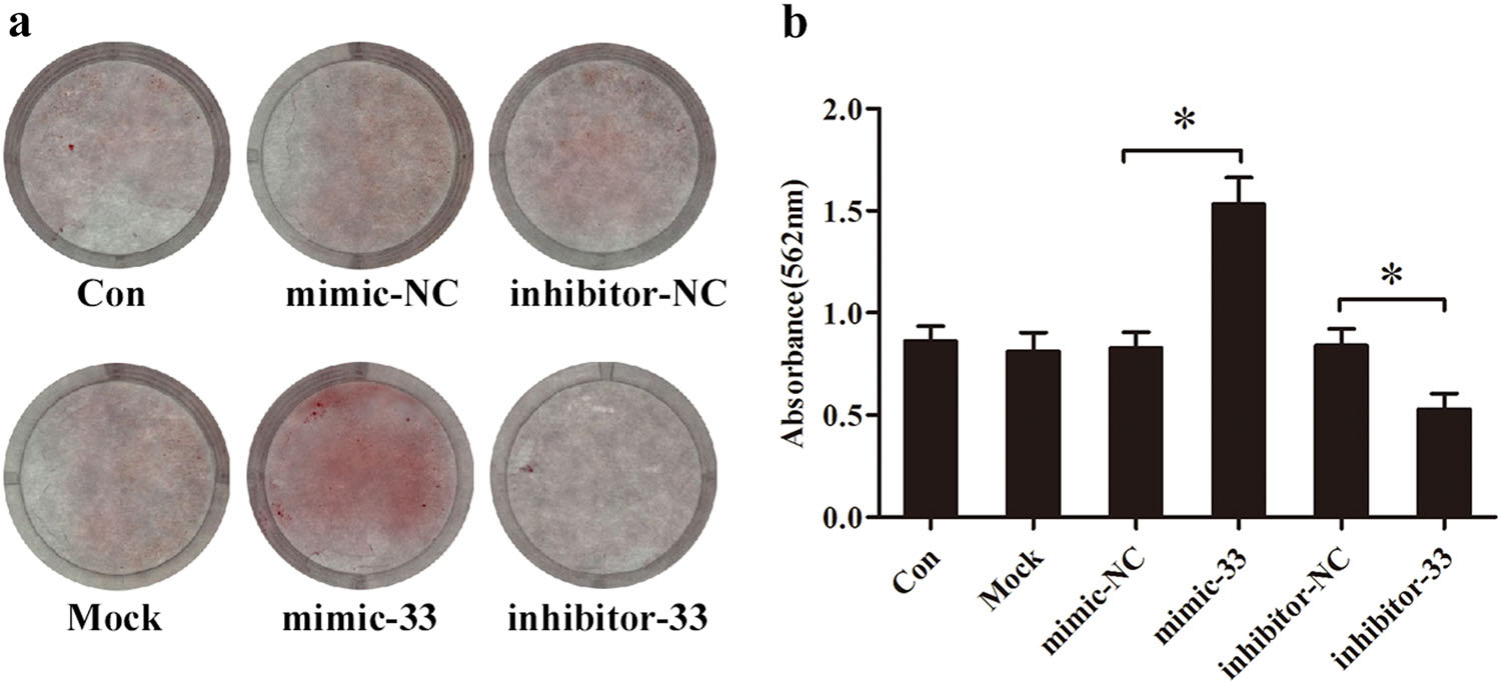
miR-33-5p promotes the mineralization of MC3T3-E1 cells. **a** Staining of calcium deposition by Alizarin red in MC3T3-E1 cells after treatment with mimic-33, inhibitor-33, or their negative controls in osteogenic medium for 21 days. **b** The absorbance of the released Alizarin red from each group at 562 nm wavelength. The data are expressed as the mean ± SD of three replicates each. *P < 0.05 vs. the negative control

In addition, we found more mineral deposition in mimic-33-treated cells and less mineral deposition in inhibitor-33-treated cells than in each corresponding control treatment group (Fig. 3a). For the quantitative analysis, we eluted the Alizarin red stain and assessed it using a microplate reader. The absorbance of the eluent solution exhibited the same pattern as the results of Alizarin red staining (Fig. 3b).

### miR-33-5p partially counteracts the decrease in osteoblast activity in HU mice

**Fig. 4:**
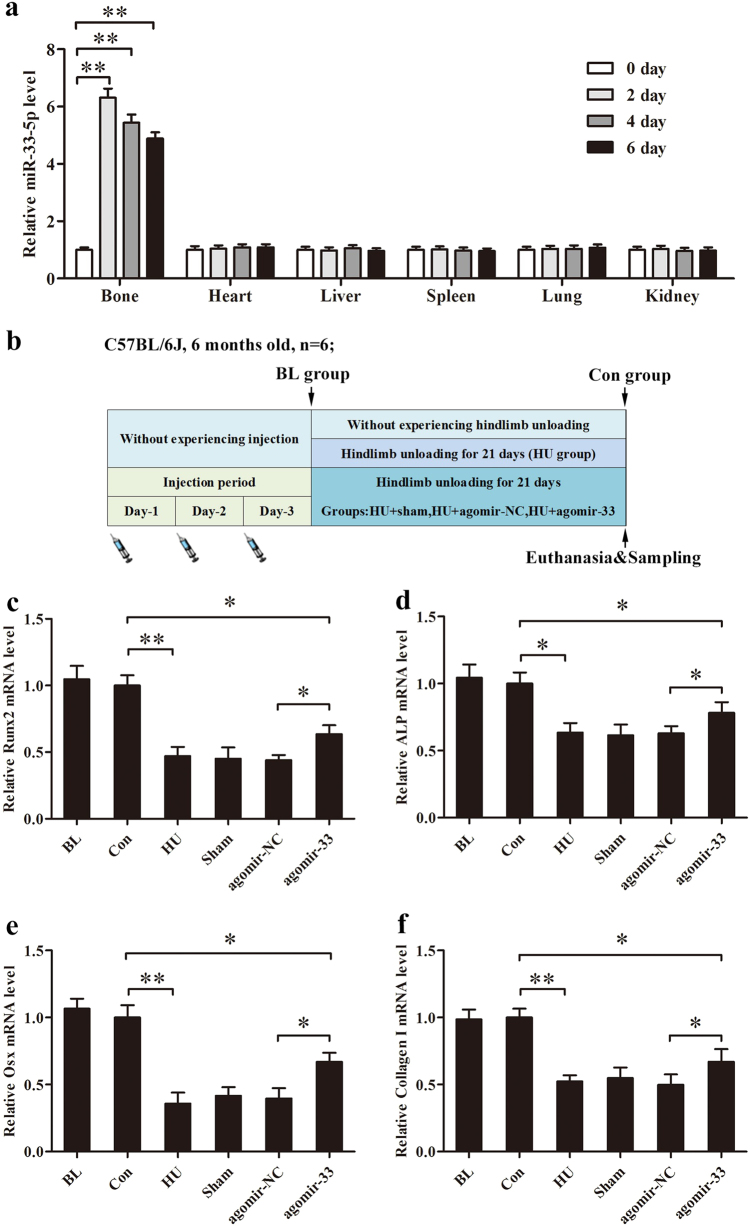
miR-33-5p partially attenuates the reduction of osteoblast differentiation makers in HU mice. **a** qRT-PCR analysis of the miR-33-5p expression in different tissues after a single injection of osteoblast-targeted agomir-33 or its negative control at different times. The values are shown relative to those of the 0 day groups. **b** A schematic diagram illustrating the experimental design. **c**–**f** qRT-PCR analysis of the changes in the mRNA expression levels of Runx2, ALP, Osx, and Collagen I in the femurs of HU mice after treatment with osteoblast-targeted agomir-33 or its negative control. The values are shown relative to that of control group. The data are expressed as the mean ± SD of six replicates each. *P < 0.05, **P < 0.01 vs. the control or negative control

Because we found that miR-33-5p could play an important role in osteoblast maturation and mineralization in vitro, we hypothesized that therapeutic supplementation with miR-33-5p in osteoblasts could counteract the decrease in bone formation in the HU mouse model as well. An osteoblast-targeted delivery system was used to deliver agomir-33 or its negative control to osteoblasts in vivo. After a single intravenous injection of agomir-33 with the osteoblast-targeted delivery system, the level of miR-33-5p was significantly increased only in the bone tissues, while in the other main tissues there were no apparent changes (Fig. 4a). This result demonstrated that the delivery system can deliver the agomir-33 to bone tissue effectively and specifically. Then we gave the mice three consecutive intravenous injections of agomir-33 with the delivery system (agomir-33 group), agomir-NC (agomir-NC group), or the delivery system only (Sham group) before HU (Fig. 4b).

**Fig. 5:**
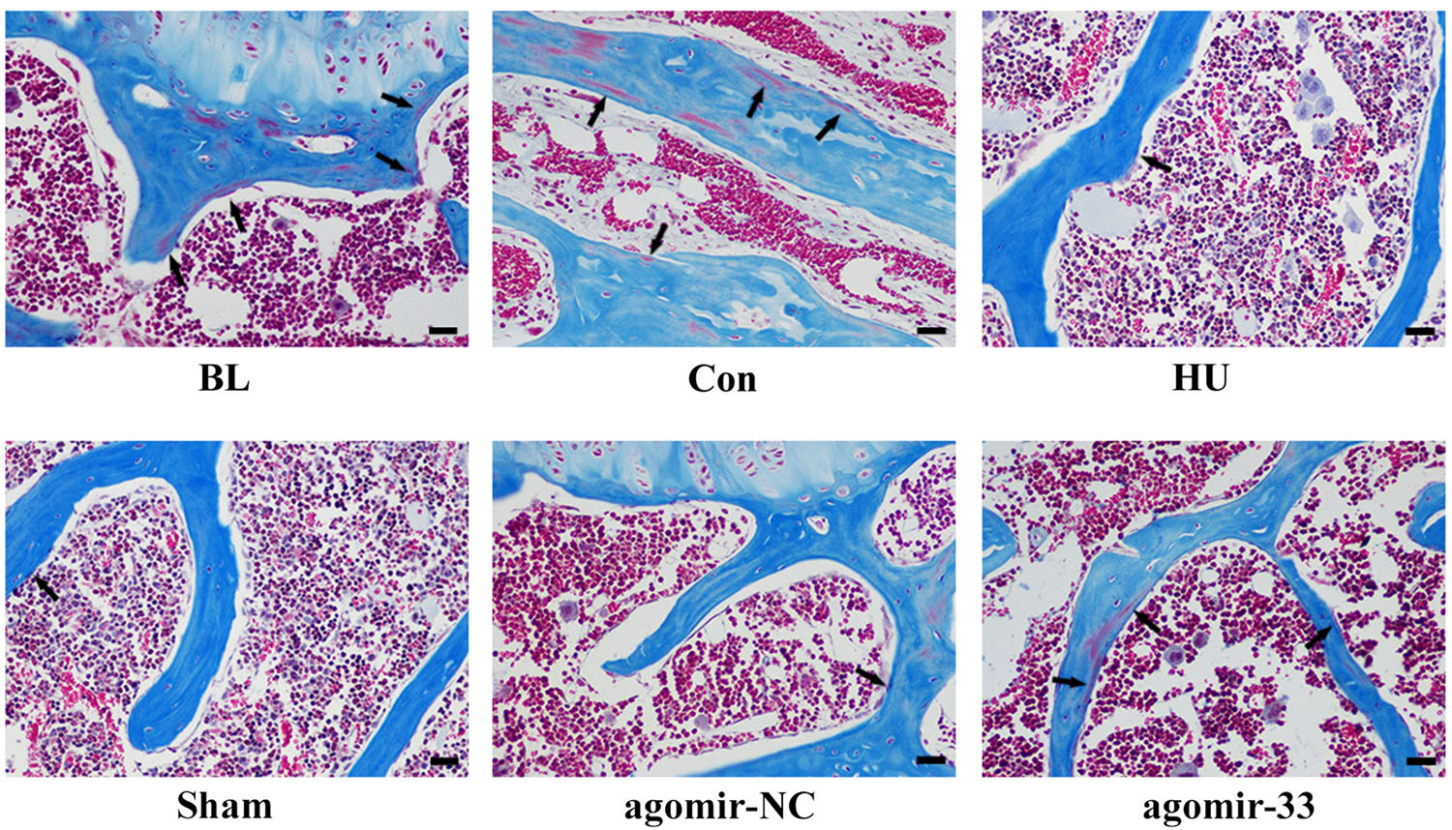
miR-33-5p displays protective effects on the bone morphology of distal femurs by Masson’s trichrome staining. Representative Masson’s trichrome staining images of distal femurs in the groups of mice indicated. Scale bar, 25 µm. Arrows indicate osteoid

We chose the mRNA levels Runx2, ALP, Osx, and Collagen I as the indicators of osteoblast differentiation and activity in vivo. The results showed that the mRNA levels of the four proteins in the femurs of the HU group mice were significantly lower than those in the control group and had no significant differences compared with the Sham and agomir-NC groups. The femurs of the agomir-33 group mice had higher mRNA levels for the four proteins than the femurs of the agomir-NC group but did not reach the level of the control group (Fig. 4c–f). All the results indicated that miR-33-5p partially counteracts the downregulated Runx2, ALP, Osx, and Collagen I mRNA expression caused by HU. Furthermore, we observed histomorphometric changes in the distal femurs. The results of Masson staining showed less osteoid staining in the distal femur from the HU group compared to that of the Con group. And compared with the agomir-NC group, osteoid were partially restored in the agomir-33 group (Fig. 5).

### miR-33-5p partially counteracts the decrease of new bone formation in HU mice

**Fig. 6:**
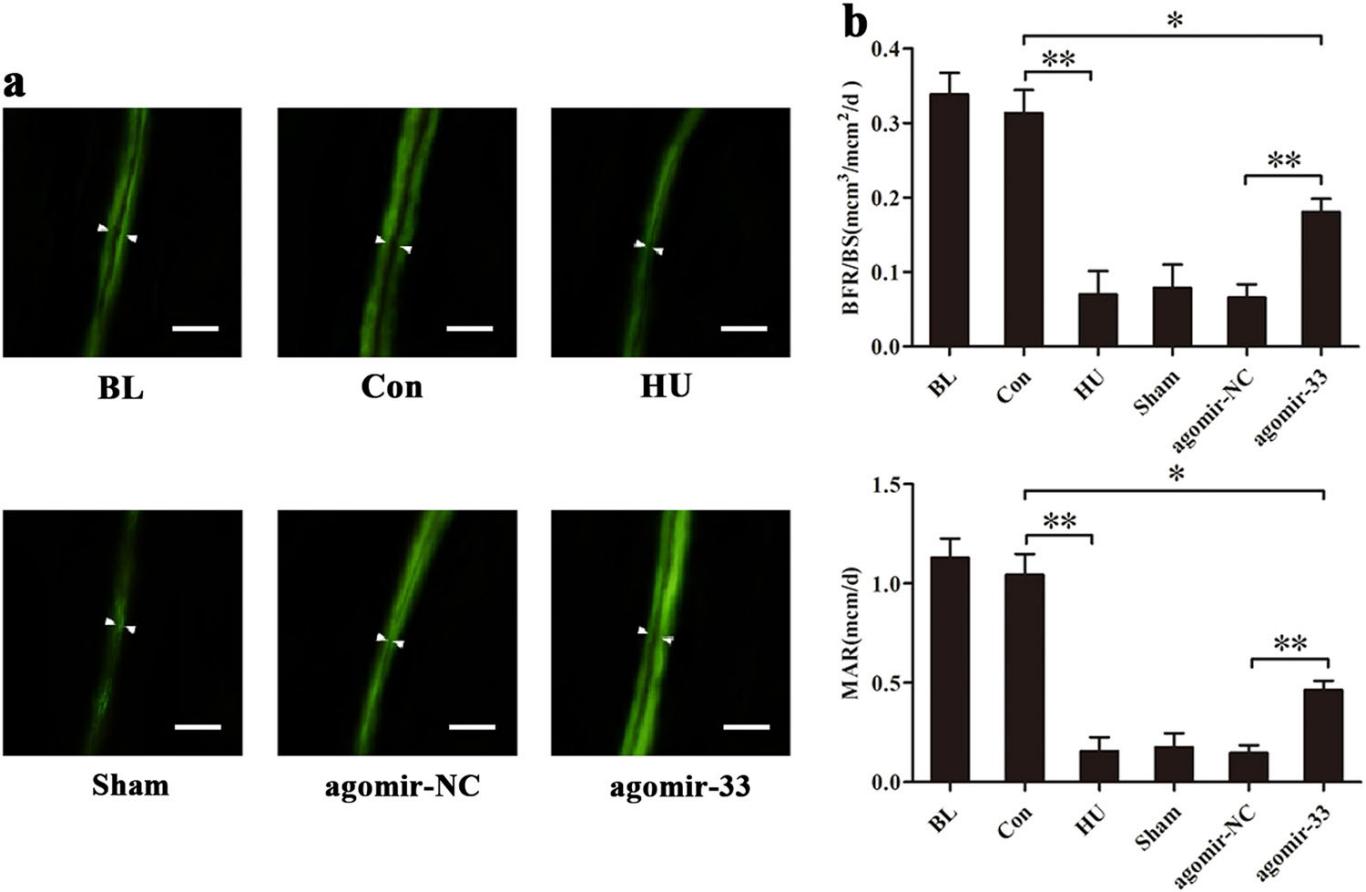
miR-33-5p displays protective effects on the mineral apposition rate of distal femurs by double calcein labeling. **a** Representative images of new bone formation assessed by double calcein labeling. Scale bar, 50 µm. **b** Histomorphometric analysis of MAR and BFR/BS in the distal femurs collected from the groups of mice indicated. The data are expressed as the mean ± SD of six replicates each. *P < 0.05, **P < 0.01 vs. the control or negative control

As we know, new bone formation is the ultimate embodiment of osteoblast function. After investigation of the protective effect of miR-33-5p on osteoblast activity in HU mice, we further explored whether it performed the same function in new bone formation. Thus we analyzed the effectiveness of new bone formation at the distal femurs by calcein double labeling. The results showed that the widths between the two fluorescence-labeled lines in the HU, Sham, and agomir-NC groups were much narrower than in the Con group. The labeled width in the agomir-33 group was slightly wider (Fig. 6a). Further measuring and calculation of bone histomorphometric parameters found that the mineral apposition rate (MAR) and the ratio of bone formation rate to bone surface (BFR/BS) were significantly decreased in the HU group compared with the Con group but had no significant differences with the Sham or the agomir-NC groups. These two parameters were significantly restored in the agomir-33 group compared with the agomir-NC group but did not reach the level of the control group (Fig. 6b).

### miR-33-5p partially counteracts the decrease in bone architecture and mechanical properties in HU mice

**Fig. 7:**
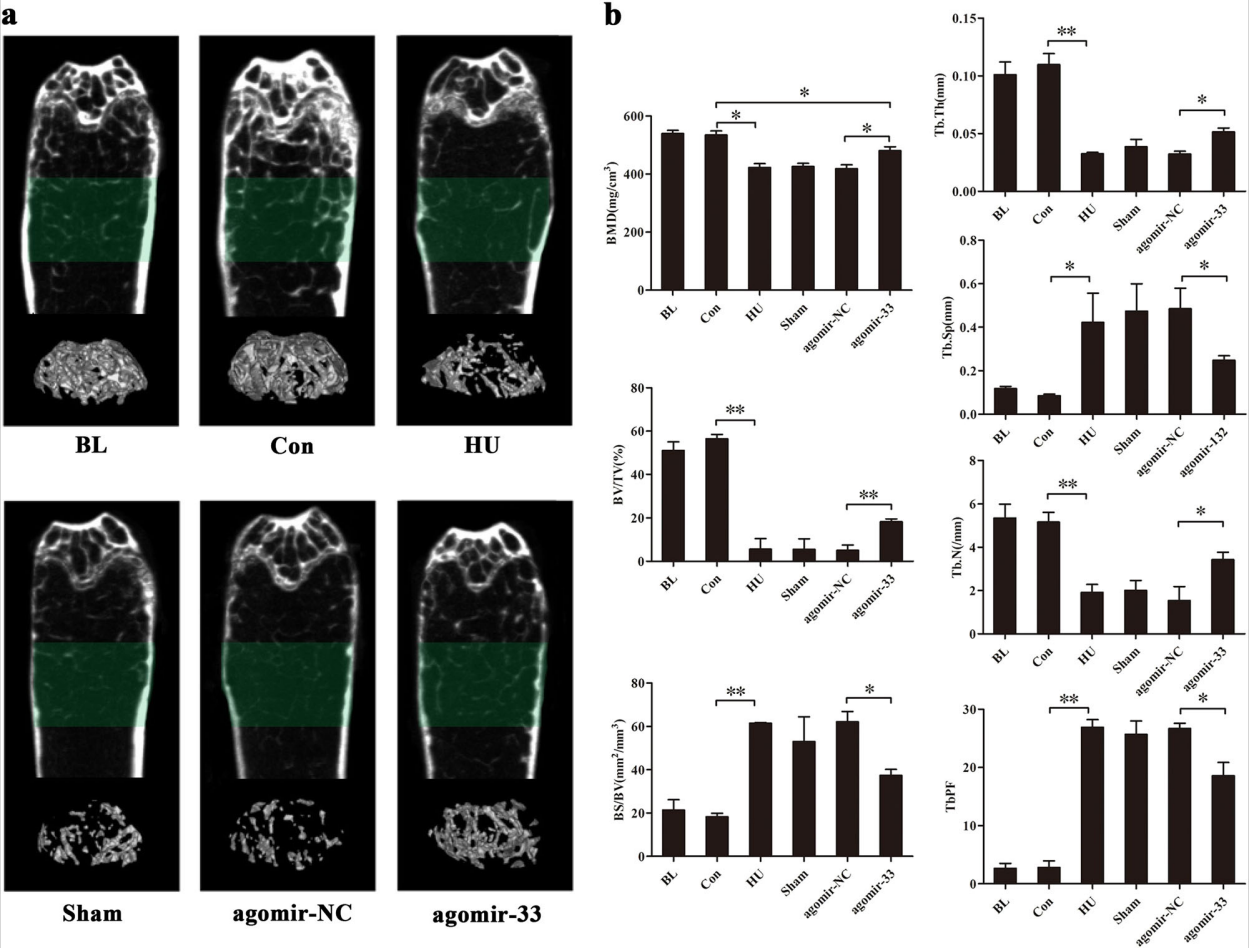
Protection by miR-33-5p on the bone mass and micro-architecture of trabecular bone of HU mice. **a** Micro-CT analysis within the distal femur of groups of mice indicated. The defined region of interest (ROI) were marked by green. The bottom row of images are the three dimensions reconstruction of the corresponding ROI. **b** Micro-CT analysis quantification within the distal femur region. The following 3D indices in the ROI were analyzed: bone mineral density (BMD), bone volume over total volume (BV/TV), bone surface over bone volume (BS/BV), trabecular thickness (Tb.Th), trabecular separation (Tb.Sp), trabecular number (Tb.N), and trabecular bone pattern factor (TbPF). The data are expressed as the mean ± SD of six replicates each. *P < 0.05, **P < 0.01 vs. the control or negative control

After testing the effect of miR-33-5p on new bone formation in HU mice, we further explored its effect on bone structure. To quantitatively analyze the bone mass and trabecular architecture precisely, we performed micro-computed tomographic (micro-CT) scanning of the distal femurs in each group. The two- and three-dimensional imaging reconstruction showed that the trabecular architecture was seriously damaged in the HU, Sham, and agomir-NC groups, while that in the agomir-33 group was intact compared with the agomir-NC group (Fig. 7a). Seven parameters were used to illustrate trabecular architecture: bone mineral density (BMD), bone volume over total volume (BV/TV), trabecular number (Tb.N), trabecular thickness (Tb.Th), bone surface over bone volume (BS/BV), trabecular separation (Tb.Sp), and trabecular bone pattern factor (TbPF). Analysis of the distal femur demonstrated that HU deteriorated the trabecular architecture, as evaluated by a decrease in BMD, BV/TV, Tb.N, and Tb.Th compared with the control group. BS/BV, Tb.Sp, and TbPF displayed a significant increase attributed to HU. Compared with the agomir-NC group, BMD, BV/TV, Tb.N, and Tb.Th were significantly increased, and BS/BV, Tb.Sp, and TbPF were significantly decreased in the agomir-33 group but did not reach the level of the control group (Fig. 7b).

**Fig. 8:**
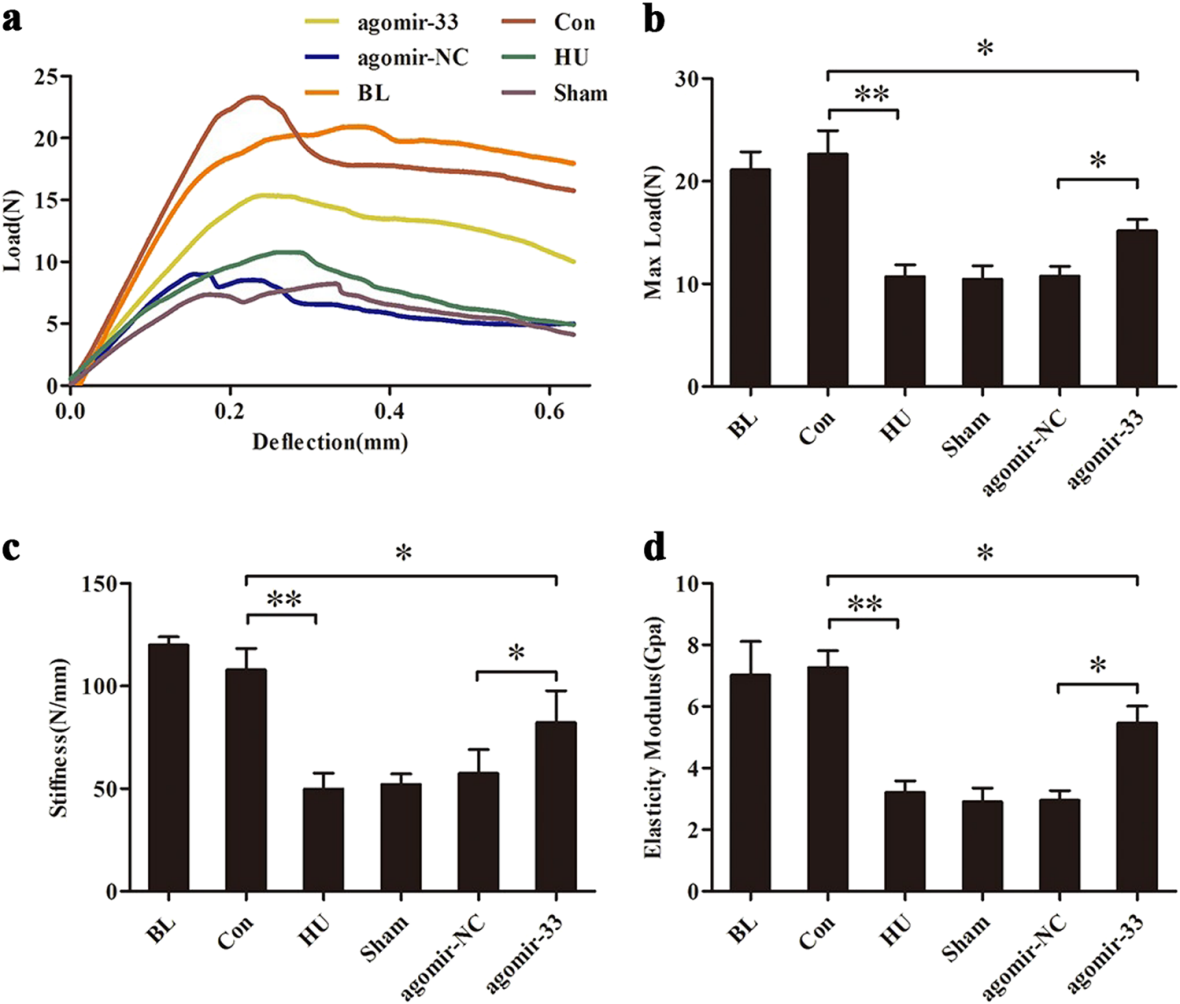
Protection by miR-33-5p on the femurs biomechanical property of HU mice measured by three-point bending test. **a** The load-deflection curves of representative samples from each groups. **b**–**d** The biomechanical property indices (max load, stiffness, and elasticity modulus) of the femurs collected from the groups of mice indicated. The data are expressed as the mean ± SD of six replicates each. *P < 0.05, **P < 0.01 vs. the control or negative control

The mechanical property of the femurs was measured by the three-point bend test. According to the data, we drew the load-deflection curves of samples in each group (Fig. 8a). Based on the load-deflection curves, we further calculated three biomechanics parameters: max load, stiffness, and elasticity modulus. The results showed that these three parameters were significantly decreased in the HU group compared with the Con group and had no significant differences with the Sham or the agomir-NC groups. While these parameters partially recovered in the agomir-33 group, they did not reach the level of the control group (Fig. 8b–d).

## Discussion

An increasing number of studies have found that miRNAs play an important role in mechanically induced bone change^35–37^. Our previous findings first demonstrated that miR-33-5p could upregulate osteoblast differentiation and partially attenuate the inhibition of osteoblast differentiation by mechanical unloading in vitro^21^. In the present study, we tested the effect of miR-33-5p on osteoblast activity, mineralization, and proliferation in vitro and found that miR-33-5p could boost the activity and mineralization of the osteoblast without influencing its proliferation. Moreover, we further investigated the anti-osteopenia effect of miR-33-5p in vivo. To improve specificity and efficiency, we used the (AspSerSer)_6_-liposome osteoblast-targeted delivery system to specifically increase the level of miR-33-5p in osteoblasts. It had an impressive therapeutic effect on mechanical unloading-induced osteopenia at bone formation, construction, and biomechanics. This study shows for the first time, to our knowledge, that miR-33-5p promotes osteoblast mineralization and it could act as a therapeutic target in mechanical unloading-induced osteopenia.

miR-33 is a miRNA family that is highly conserved from Drosophila to humans. Two isoforms of miR-33, miR-33a and miR-33b, are expressed in humans. However, only one miR-33 isoform, miR-33a, is expressed in mice and conserved in humans. Human miR-33a has two subtypes, miR-33a-3p and miR-33a-5p, which correspond to miR-33-3p and miR-33-5p in mice, respectively^38,39^. miR-33-5p was first reported in 2007, where miR-33 was attenuated by the downregulation of FoxG1 expression during forebrain development^40^. Subsequent studies further verified that miR-33a, an intronic microRNA located within the SREBF-2 gene, plays a key role in the homeostatic regulation of cholesterol metabolism^41^. Moreover, miR-33a expression in both macrophages and hepatocytes has been found to be inversely correlated with cholesterol level. Knockdown of miR-33 also promotes cholesterol trafficking in vitro and high-density lipoprotein synthesis in vivo^42^. Many studies subsequently performed antisense therapeutic targeting of miR-33 in individuals suffering from cardiometabolic diseases^11,43–47^. There has also been some exploration of the regulatory effect of miR-33 in oncology^48–50^. miR-33 was upregulated in human papillomavirus-positive cases of squamous cell carcinoma of the head and neck, while downregulated in biopsies from myotonic dystrophy type-1 patients^51^. In addition, one study found that overexpression of miR-33a in A375 cells significantly inhibited melanoma tumorigenesis, identifying miR-33 as a tumor suppressor in melanoma^52^. Furthermore, there are some other important functions of miR-33, such as regulating cellular energy sensing, mitochondrial biogenesis, and mitochondrial fatty acid oxidation^53–55^. Impressively, our previous results extend these earlier findings by demonstrating that miR-33-5p also regulates osteoblast differentiation in MC3T3-E1 cells. In addition, it could sense multiple mechanical environments, both contact (flow shear stress) and non-contact forces (gravity), in MC3T3-E1 cells in vitro. Our previous data also show that overexpression of miR-33-5p increases the expression of Runx2, Osx, and ALP, indicating that miR-33-5p promotes osteoblast differentiation in vitro. Moreover, flow shear stress and the microgravity environment both affect osteoblast differentiation by modulating miR-33-5p. Specifically, miR-33-5p functions by inhibiting its direct target, Hmga2, at the posttranscriptional level to negatively affect the differentiation of MC3T3-E1 cells. The present data further indicated that miR-33-5p positively regulated the osteoblastic activity of MC3T3-E1 cells, using OCN and Collagen I as markers. Moreover, it also positively regulated the mineralization of MC3T3-E1 cells without influencing their proliferation. These data filled research gaps in the regulatory effect of miR-33-5p on osteoblasts in vitro.

Osteoporosis, a common disease, is treated with some well-understood medications, such as diphosphonate, calcitonin, and vitamin D. To pursue better curative effects, researchers are exploring new mechanisms and therapeutic targets of different kinds of osteoporosis^56^. With the importance of miRNA receiving much more attention, miRNA is becoming a novel and effective therapeutic target in osteoporosis. Some studies have tried to confirm that modulating miRNAs could combat osteoporosis. miR-27a was found to be decreased in mesenchymal stem cells (MSCs) of ovariectomized mice, which decreased bone formation. In addition, miR-27a was found to be essential for the osteogenic differentiation of MSCs by targeting Mef2c^57^. miR-210 was proven to promote osteoblastic differentiation of MSCs by increasing the expression of ALP and Osx, and it played an important role in ameliorating postmenopausal osteoporosis through promoting vascular endothelial growth factor expression and osteoblast differentiation^58,59^. Furthermore, there are studies that explored the protective effect of miRNAs to osteopenia induced by mechanical unloading. In mechanical stretch-induced osteoblast differentiation, miR-103a was significantly decreased and negatively regulated its directly target gene (Runx2). Therapeutic inhibition of miR-103a partly rescued the osteopenia caused by mechanical unloading in vivo^36^. However, a single injection of miRNA modulators still has some disadvantages that cannot be ignored, such as side effects in other tissues, rapid biodegradation, amplification of experimental cost, etc. Targeted delivery systems were invented and applied in multiple research areas to solve these problems. The (AspSerSer)_6_-liposome delivery system was invented and first reported in 2012. It successfully delivered the siRNA of Plekho1, a specific osteoblastic inhibitor, to the osteoblasts in the bone formation area and then effectively relieved the simulated primary osteoporosis in vivo^35^. Next, this delivery system was used to target the delivery of the antagomir of miR-214 and acquired an anti-osteoporosis effect in both ovariectomized and hindlimb-unloaded mice^17^. In the present study, we used the new strategy for osteoporosis interference, miRNA-targeted treatment, and used a delivery system to ensure high specificity and efficiency. Most recent studies of miRNA-targeted treatment adopted the method of inhibiting the target miRNA, while we found that supplementation with a miRNA could also resist osteoporosis.

Tail vein injection is a common and efficient method to administer drugs in mice. However, because of the limitations of the HU animal model, it is not possible to administer tail vein injections during HU. Therefore, we gave the mice three consecutive intravenous injections of agomir-33 with the delivery system once a day before unloading. Prior to the final experiment, we carried out a pretest and verified that the (AspSerSer)_6_-liposome delivery system could deliver the agomir-33 to bone tissue specifically. We also showed that a single injection of agomir-33 with the delivery system could maintain the increased miR-33-5p level in bone tissue ( > 3-fold) over 6 days. Our observation is consistent with data from pharmacodynamic tests showing that administering a siRNA with this delivery system could maintain a low target gene mRNA level (<50% of baseline mRNA level) for approximately 7 days^34^. However, we tested the miR-33-5p levels in the femurs of each group at the end of the experiments and found no significant differences in miR-33-5p levels between the agomir-33 group, the agomir-NC group, and the Sham group (Supplementary Fig. 2). Because of the limitations of the experimental materials, we could not test the time course of changes in miR-33-5p levels during HU. However, it can be speculated that the time of effectively upregulated miR-33-5p level by the three consecutive injections could be substantially longer than 6 or 7 days, which is certainly satisfactory for the experiment requirements.

Depending on the high binding affinity of (AspSerSer)_6_ to lowly crystallized hydroxyapatite at the bone-formation surface, the delivery system could bring the agomir-33 to the bone-formation surface enriched in osteoblasts^17,35^. Then agomir-33 entered the osteoblasts to increase the miR-33-5p level, which was mediated by the liposome. Our results showed that targeting supplementation with miR-33-5p partially recovered the inhibition of osteoblast activity induced by mechanical unloading in vivo. By promoting the activity of osteoblasts in vivo, supplementation with miR-33-5p enhanced the bone-formation ability and increased the osteoid formation, further increasing the thickness and width of new bone. Next, by enhancing the ability of bone formation, supplementation with miR-33-5p further improved the trabecular structure that was seriously damaged by mechanical unloading and enhanced the biomechanical properties of the femurs. Based on our finding that miR-33-5p promotes osteoblast activity in vitro, our data further demonstrated the protective effect of miR-33-5p to mechanical unloading-induced osteopenia from a variety of aspects in vivo.

It should be noted that there are some limitations to our study. In our previous study, we verified that miR-33-5p promoted osteoblast differentiation by targeting the inhibition of Hmga2 proteins. In the present study, we observed a regulatory effect of miR-33-5p on bone formation but lacked research into the Hmga2 mechanism in vivo. We are working to acquire a sufficient number of osteoblast-specific Hmga2 knock-out and knock-in mice to further verify the existence of the miR-33-5p/Hmga2 pathway in vivo. In addition, due to the experimental material constraints, we tested the markers of osteoblast activity at the mRNA level, and we would further test these markers at the protein level using western blotting and immunohistochemistry in a future study.

In summary, our study provides a new finding that miR-33-5p promotes osteoblast activity in vitro and insignificantly influences osteoblast proliferation. Furthermore, supplementation with miR-33-5p in osteoblasts by a targeted delivery system partially recovered the osteopenia induced by mechanical unloading. Our findings further enriched the mechanisms of miRNA in bone research and provided a new molecular target therapeutic strategy for osteopenia.

## Materials and methods

### Cell culture and transfection

MC3T3-E1 cells, a mouse pre-osteoblast cell line, were purchased from the Cell Bank of the Chinese Academy of Sciences (Shanghai, China). The MC3T3-E1 cells were maintained in Dulbecco’s modified Eagle’s medium F12 1:1 medium (HyClone, Utah, USA) with 10% fetal bovine serum (HyClone, Utah, USA) and 1% penicillin and streptomycin (HyClone, Utah, USA). The cells were incubated at 37.0 °C culture conditions of 5% CO_2_ and 95% humidity. The cells were subcultured every 72 h and used at passages 8–12. To induce osteoblastic differentiation for the functional tests, MC3T3-E1 cells were maintained in osteogenic medium containing 10 mM β-glycerophosphate, 50 μM ascorbic acid, and 100 nM dexamethasone (Sigma-Aldrich, Missouri, USA). Confluent cells were removed using 0.25% trypsin containing 10 mM EDTA (HyClone, Utah, USA), resuspended in antibiotic-free growth medium, and plated onto 6- or 96-well plates at a density of 1.0 × 10^5^/cm^2^.

For transfection of miRNA modulator oligomers, the medium transfected by Lipofectamine^TM^ 2000 (Invitrogen, California, USA) was used according to the manufacturer’s instructions. The mimic or inhibitor of miR-33-5p and their negative controls (RiboBio, Guangzhou, China) were transfected at the concentration of 100 nM.

### Western blot analysis

Cells were lysed using RIPA buffer (Beyotime Biotechnology, Shanghai, China) containing a protease inhibitor (Beyotime Biotechnology, Shanghai, China). Equal amounts of protein from each sample were added to a NuPage Bis-Tris polyacrylamide gel (Invitrogen, California, USA) and run for 2 h using MES SDS running buffer (Invitrogen, California, USA). The proteins were then transferred to nitrocellulose membranes, which were blocked for 5 h at room temperature with milk (5% w/v) in Tris-buffered saline (TBS) containing Tween-20 (0.1%; TBS-T). The blots were subsequently incubated overnight with a primary antibody (1:2000) against Collagen I or OCN at 4 °C with oscillation, after which they were incubated with a horseradish peroxidase-conjugated secondary antibody (1:10,000; Jackson, USA). The secondary antibodies were detected and visualized using the Super Signal West substrate (Fisher Scientific, Massachusetts, USA). The resulting bands were quantified through densitometry with the ImageJ software using the Collagen I antibody (Abcam, ab34710, USA) and the OCN antibody (Abcam, ab93876, USA).

### Quantitative reverse transcriptase PCR (qRT-PCR)

Total RNA from bone tissues or cells was extracted with the TRIzol Reagent (Invitrogen, California, USA) according to the manufacturer’s instructions. First-strand cDNA was synthesized by incubating 1 μg of total RNA with Superscript III reverse transcriptase (Takara, Tokyo, Japan) for 1 h at 42.0 °C following oligo (dT) priming. qRT-PCR was performed using the CFX96 (BIO-RAD, California, USA) instrument and individual PCRs were performed in 96-well optical reaction plates with SYBR Green-I (Takara, Tokyo, Japan) according to the manufacturer’s instructions. Target gene expression was normalized to that of the reference gene GAPDH. The 2^−ΔΔCt^ method was used to calculate the relative gene expression. These PCR products were subjected to melting curve analysis and a standard curve to confirm the correct amplification. All PCRs were performed in triplicate, and the primers used for PCR are listed in Supplementary Table 1.

### Immunofluorescence

Cultured cells were gently washed with cold phosphate-buffered saline (PBS) to remove nonadherent cells before fixation in 2.0% paraformaldehyde for 15 min at room temperature. Then cells were permeabilized in 0.025% Triton X-100 in PBS for 10 min before incubation for 1 h with normal goat serum. After the introduction of diluted primary antibody to the cells overnight at 2–8 °C, the cells were incubated with secondary antibody fluorescein isothiocyanate (Abcam, ab7086, USA) diluted in blocking solution in a dark humidity chamber for 1 h. The sample was counterstained with 4,6-diamidino-2-phenylindole at room temperature for 10 min, and the slides were stored in the dark at 2–8 °C. The micrographs were obtained by laser scanning confocal microscopy (Olympus FV1000, Tokyo, Japan).

### Cell proliferation assay

Cell proliferation was evaluated using the Cell Counting Kit-8 (WST-8) assay (Dojindo, Tokyo, Japan). The cells were seeded into 96-well plates at a density of 5 × 10^3^ cells/well in 200 μl medium. The WST-8 assay was performed following the manufacturer’s protocol for 24–96 h. In brief, WST-8 solution was added to the culture medium, and the cells were incubated at 37 °C and 5% CO_2_ for an additional 3.5 h. The absorbance of the reaction solution at 450 nm was measured by a microplate reader (Molecular Devices, California, USA).

### Alizarin red staining

Cells were fixed in 90% cold ethanol for 10 min and gently rinsed with ddH_2_O. Cells were stained with 40 mM Alizarin red S (Sigma-Aldrich, Missouri, USA) with a pH of 4.0 for 15 min with gentle agitation. Cells were rinsed three times with ddH_2_O and then rinsed for 15 min with DPBS while gently agitating.

### HU model

The animal model of mechanical unloading-induced osteopenia was copied in HU mice. Male C57BL/6j mice at 6 months of age were individually caged and suspended by the tail using a strip of adhesive surgical tape attached to a chain hanging from a pulley under standard conditions (12 h light/12 h dark cycle, 21.0 °C controlled temperature). The mice were suspended at an approximately 30° angle to the floor with only the forelimbs touching the floor. This allowed the mice to freely move and access to food and water. Three consecutive injections once a day were executed before HU. The agomir-33 (or the negative control) and the (AspSerSer)_6_-liposome delivery system were well mixed before use, as described previously. The mice were anesthetized after 3 weeks of tail suspension. After euthanasia, bilateral femurs and tibiae were dissected and processed for micro-CT examination, bone histomorphometric analysis, and quantitative real-time PCR (qRT-PCR) analysis. All the experimental procedures were approved by the Committees of Animal Ethics and Experimental Safety of the Chinese Academy of Sciences.

### Micro-CT analysis

The right femur of each rat was fixed in 4% paraformaldehyde for 24 h and then scanned using micro-CT (Siemens, Bavaria, Germany). The scanning energy was set at 80 kv and 500 mA. The sample was scanned over a 360° rotation with an exposure time of 800 ms/frame at a resolution of 10.44 μm. The angle of increment around the sample was set to 0.5°. A 2.5 × 2.5 × 3 mm^3^ cube approximately 1.5 mm away from the proximal epiphyseal growth plate was selected as the region of interest (ROI) to represent the microstructure of the femur.

The following 3D indices in the defined ROI were analyzed: BMD, BV/TV, Tb.N, Tb.Th, and Tb.Sp. The operator who performed the scan analysis was blinded to the treatment associated with the specimens.

### Three-point bending test

To measure bone strength, a three-point bending test was performed using an electromechanical material-testing machine (Bose, Massachusetts, USA) set at span length of 8 mm and a loading speed of 0.02 mm/s. Load was applied to the anterior surface of the diaphyseal mid-part, aiming at applying load at the same site. Load and deformation data were recorded and sampled at 50 Hz. The load deflection curves were used to calculate max load at failure (N), stiffness (slope of the load-deflection curve, representing the elastic deformation, N/mm), and elasticity modulus (Gpa).

### Masson staining

The fixed femurs were decalcified by decalcifying solution with EDTA (Beyotime Biotechnology, Shanghai, China). After dehydration, the samples were embedded in paraffin, and 4 μm sections were prepared on a rotation microtome (Thermo, Massachusetts, USA). Bone sections were Masson stained following the manufacturer’s protocol (Sigma-Aldrich, Missouri, USA).

### Calcein double labeling

Calcein labeling of the bones was performed by intraperitoneal injection of calcein (5 mg/kg) administered at 10 and 3 days before euthanasia. Then the femurs were fixed, dehydrated, embedded, and sliced (50 μm). At the point of intersection, the distance between the middle of two fluorescein labels was measured by the ImageJ software. Two histomorphometric parameters, MAR and bone formation rate (BFR/BS), were calculated.

### Statistical analysis

The experimental data were statistically analyzed with the SPSS 17.0 software. The data are expressed as the mean ± SD of at least three independent experiments in vitro and six independent experiments in vivo. Comparisons were performed using a two-tailed *t*-test or one-way analysis of variance for experiments with more than two subgroups. A *P*-value of <0.05 was considered significant.

## Electronic supplementary material

Supplementary information

## References

[1] Yang M (2014). The role of integrin-beta/FAK in cyclic mechanical stimulation in MG-63 cells. Int J. Clin. Exp. Pathol..

[2] Kim DS, Jung SM, Yoon GH, Lee HC, Shin HS (2014). Development of a complex bone tissue culture system based on cellulose nanowhisker mechanical strain. Colloids Surf. B. Biointerfaces.

[3] Saito M, Soshi S, Fujii K (2003). Effect of hyper- and microgravity on collagen post-translational controls of MC3T3-E1 osteoblasts. J. Bone Miner. Res..

[4] Aisha MD, Nor-Ashikin MN, Sharaniza AB, Nawawi H, Froemming GR (2015). Orbital fluid shear stress promotes osteoblast metabolism, proliferation and alkaline phosphates activity in vitro. Exp. Cell Res..

[5] Landis WJ, Hodgens KJ, Block D, Toma CD, Gerstenfeld LC (2000). Spaceflight effects on cultured embryonic chick bone cells. J. Bone Miner. Res..

[6] Ontiveros C, McCabe LR (2003). Simulated microgravity suppresses osteoblast phenotype, Runx2 levels and AP-1 transactivation. J. Cell. Biochem..

[7] Blaber EA (2013). Microgravity induces pelvic bone loss through osteoclastic activity, osteocytic osteolysis, and osteoblastic cell cycle inhibition by CDKN1a/p21. PLoS ONE.

[8] Boehrs J, Zaharias RS, Laffoon J, Ko YJ, Schneider GB (2008). Three-dimensional culture environments enhance osteoblast differentiation. J. Prosthodont..

[9] Uddin SM, Qin YX (2013). Enhancement of osteogenic differentiation and proliferation in human mesenchymal stem cells by a modified low intensity ultrasound stimulation under simulated microgravity. PLoS ONE.

[10] Fernandez-Hernando C, Suarez Y, Rayner KJ, Moore KJ (2011). MicroRNAs in lipid metabolism. Curr. Opin. Lipidol..

[11] Najafi-Shoushtari SH (2011). MicroRNAs in cardiometabolic disease. Curr. Atheroscler. Rep..

[12] Suarez Y, Fernandez-Hernando C (2016). Preface to: “microRNAs in lipid/energy metabolism and cardiometabolic disease”. Biochim. Biophys. Acta.

[13] Wang Y (2015). Mechanical strain affects some microrna profiles in pre-oeteoblasts. Cell. Mol. Biol. Lett..

[14] Liao L (2013). Redundant miR-3077-5p and miR-705 mediate the shift of mesenchymal stem cell lineage commitment to adipocyte in osteoporosis bone marrow. Cell Death Dis..

[15] Mai ZH (2013). miRNA expression profile during fluid shear stress-induced osteogenic differentiation in MC3T3-E1 cells. Chin. Med. J. (Engl.).

[16] Palmieri A (2007). Differences in osteoblast miRNA induced by cell binding domain of collagen and silicate-based synthetic bone. J. Biomed. Sci..

[17] Wang X (2013). miR-214 targets ATF4 to inhibit bone formation. Nat. Med..

[18] Sun Z (2015). Simulated microgravity inhibits L-type calcium channel currents partially by the up-regulation of miR-103 in MC3T3-E1 osteoblasts. Sci. Rep..

[19] Sun Z (2015). MiR-103 inhibits osteoblast proliferation mainly through suppressing Cav1.2 expression in simulated microgravity. Bone.

[20] Hu Z (2015). miRNA-132-3p inhibits osteoblast differentiation by targeting Ep300 in simulated microgravity. Sci. Rep..

[21] Wang H (2016). miR-33-5p, a novel mechano-sensitive microRNA promotes osteoblast differentiation by targeting Hmga2. Sci. Rep..

[22] Akao Y (2011). Microvesicle-mediated RNA molecule delivery system using monocytes/macrophages. Mol. Ther..

[23] Hauser B (2015). Functions of MiRNA-128 on the regulation of head and neck squamous cell carcinoma growth and apoptosis. PLoS ONE.

[24] Li Y (2013). The promotion of bone regeneration through positive regulation of angiogenic-osteogenic coupling using microRNA-26a. Biomaterials.

[25] Tseng YC, Huang L (2009). Self-assembled lipid nanomedicines for siRNA tumor targeting. J. Biomed. Nanotechnol..

[26] Zhang X (2012). Analytical methods for brain targeted delivery system in vivo: perspectives on imaging modalities and microdialysis. J. Pharm. Biomed. Anal..

[27] Ando H (2013). Polycation liposomes as a vector for potential intracellular delivery of microRNA. J. Gene Med..

[28] Kim DG (2016). Anti-miR delivery strategies to bypass the blood-brain barrier in glioblastoma therapy. Oncotarget.

[29] Zhang Y, Buhrman JS, Liu Y, Rayahin JE, Gemeinhart RA (2016). Reducible micelleplexes are stable systems for Anti-miRNA delivery in cerebrospinal fluid. Mol. Pharm..

[30] Janssen HL (2013). Treatment of HCV infection by targeting microRNA. N. Engl. J. Med..

[31] Hsu SH (2013). Cationic lipid nanoparticles for therapeutic delivery of siRNA and miRNA to murine liver tumor. Nanomedicine.

[32] Ando H (2013). Development of a miR-92a delivery system for anti-angiogenesis-based cancer therapy. J. Gene Med..

[33] Liang C (2015). Aptamer-functionalized lipid nanoparticles targeting osteoblasts as a novel RNA interference-based bone anabolic strategy. Nat. Med..

[34] Zhang G (2012). A delivery system targeting bone formation surfaces to facilitate RNAi-based anabolic therapy. Nat. Med..

[35] Zuo B (2015). microRNA-103a functions as a mechanosensitive microRNA to inhibit bone formation through targeting Runx2. J. Bone Miner. Res..

[36] Cao Y, LV Q, LV C (2015). MicroRNA-153 suppresses the osteogenic differentiation of human mesenchymal stem cells by targeting bone morphogenetic protein receptor type II. Int. J. Mol. Med..

[37] Wei FL (2014). Mechanical force-induced specific MicroRNA expression in human periodontal ligament stem cells. Cells Tissues Organs.

[38] Weber MJ (2005). New human and mouse microRNA genes found by homology search. FEBS J..

[39] Chiang HR (2010). Mammalian microRNAs: experimental evaluation of novel and previously annotated genes. Genes Dev..

[40] Bredenkamp N, Seoighe C, Illing N (2007). Comparative evolutionary analysis of the FoxG1 transcription factor from diverse vertebrates identifies conserved recognition sites for microRNA regulation. Dev. Genes Evol..

[41] Najafi-Shoushtari SH (2010). MicroRNA-33 and the SREBP host genes cooperate to control cholesterol homeostasis. Science.

[42] Horie T (2010). MicroRNA-33 encoded by an intron of sterol regulatory element-binding protein 2 (Srebp2) regulates HDL in vivo. Proc. Natl. Acad. Sci. USA.

[43] Fernandez-Hernando C, Ramirez CM, Goedeke L, Suarez Y (2013). MicroRNAs in metabolic disease. Arterioscler. Thromb. Vasc. Biol..

[44] Cao, R. et al. Xuezhikang therapy increases miR-33 expression in patients with low HDL-C levels. *Dis. Markers* **2014**, 781780 (2014). 10.1155/2014/781780 PMC3925606 24591767

[45] Zhao GJ (2014). Chlamydia pneumoniae negatively regulates ABCA1 expression via TLR2-Nuclear factor-kappa B and miR-33 pathways in THP-1 macrophage-derived foam cells. Atherosclerosis.

[46] Vega-Badillo J (2016). Hepatic miR-33a/miR-144 and their target gene ABCA1 are associated with steatohepatitis in morbidly obese subjects. Liver Int..

[47] Goedeke L (2014). Long-term therapeutic silencing of miR-33 increases circulating triglyceride levels and hepatic lipid accumulation in mice. EMBO Mol. Med..

[48] Lin Y (2015). MicroRNA-33b inhibits breast cancer metastasis by targeting HMGA2, SALL4 and Twist1. Sci. Rep..

[49] Wolfe AR (2016). MiR-33a decreases high-density lipoprotein-induced radiation sensitivity in breast cancer. Int. J. Radiat. Oncol. Biol. Phys..

[50] Adam L (2013). Plasma microRNA profiles for bladder cancer detection. Urol. Oncol..

[51] Wald AI, Hoskins EE, Wells SI, Ferris RL, Khan SA (2011). Alteration of microRNA profiles in squamous cell carcinoma of the head and neck cell lines by human papillomavirus. Head Neck.

[52] Zhou J (2015). miR-33a functions as a tumor suppressor in melanoma by targeting HIF-1alpha. Cancer Biol. Ther..

[53] Price NL, Fernandez-Hernando C (2015). Novel role of miR-33 in regulating of mitochondrial function. Circ. Res..

[54] Karunakaran D (2015). Macrophage mitochondrial energy status regulates cholesterol efflux and is enhanced by anti-miR33 in atherosclerosis. Circ. Res..

[55] Ouimet M (2015). MicroRNA-33-dependent regulation of macrophage metabolism directs immune cell polarization in atherosclerosis. J. Clin. Invest..

[56] Laurent M, Gielen E, Claessens F, Boonen S, Vanderschueren D (2013). Osteoporosis in older men: recent advances in pathophysiology and treatment. Best. Pract. Res. Clin. Endocrinol. Metab..

[57] You L, Pan L, Chen L, Gu W, Chen J (2016). MiR-27a is essential for the shift from osteogenic differentiation to adipogenic differentiation of mesenchymal stem cells in postmenopausal osteoporosis. Cell. Physiol. Biochem..

[58] Liu XD, Cai F, Liu L, Zhang Y, Yang AL (2015). MicroRNA-210 is involved in the regulation of postmenopausal osteoporosis through promotion of VEGF expression and osteoblast differentiation. Biol. Chem..

[59] Liu SC (2014). CTGF increases vascular endothelial growth factor-dependent angiogenesis in human synovial fibroblasts by increasing miR-210 expression. Cell Death Dis..

