# Supplementary material for: Osteoblast-targeted delivery of miR-33-5p attenuates osteopenia development induced by mechanical unloading in mice
Source: Cell Death Dis. 2018 Feb 7;9(2):170. doi: 10.1038/s41419-017-0210-5 (PMC5833703; doi:10.1038/s41419-017-0210-5)
Supplement: Supplementary file 1 — Supplementary information [file 41419_2017_210_MOESM1_ESM.doc]

**Supplementary information**

**Supplement miR-33-5p in osteoblast by a targeting delivery system partially recovers mechanical unloading induced osteopenia in vivo.**

Han Wang^1,2*^, Zebing Hu^2*^, Shi Fei^2*^, Jingjing Dong^2*^, Lei Dang^4^, Yixuan Wang^2^, Zhongyang Sun^2,3^, Hua Zhou^2^, Shu Zhang^2^, Xinsheng Cao^2^ and Ge Zhang^4^

^1^Department of Orthopedics, Affiliated Hospital of Air Force Aviation Medicine Research Institute, The Fourth Military Medical University, 100089, Beijing, China.

^2^The Key Laboratory of Aerospace Medicine, Ministry of Education, The Fourth Military Medical University, 710032, Xi’an, Shaanxi, China.

^3^Department of Orthopedics, No. 454 Hospital of PLA, 210002, Nanjing, Jiangsu, China.

^4^Institute for Advancing Translational Medicine in Bone & Joint Diseases, School of Chinese Medicine, Hong Kong Baptist University, Hong Kong SAR, China.

**Supplementary Table 1. The sequence of primers.**

| Name | Sequence(5'-3') |
| --- | --- |
| miR-33-5p RT | CTCAACTGGTGTCGTGGAGTCGGCAATTCAGTTGAGTGCAATGC |
| miR-33-5p-F | ACACTCCAGCTGGGGTGCATTGTAGTT |
| miR-33-5p-R | TGGTGTCGTGGAGTCG |
| U6-F | CTCGCTTCGGCAGCACA |
| U6-R | AACGCTTCACGAATTTGCGT |
| GAPDH-F | CCTCTGACTTCAACAGCGAC |
| GAPDH-R | TCCTCTTGTGCTCTTGCTGG |
| Runx2-F | CCTCTGACTTCTGCCTCTGG |
| Runx2-R | ATGAAATGCTTGGGAACTGC |
| Osx-F | AGGCACAAAGAAGCCATACG |
| Osx-R | GCCCAGGAAATGAGTGAGG |
| ALP-F | CGTGGGCATTGTGACTACC |
| ALP-R | CTGGTGGCATCTCGTTATCC |
| Collagen 1a-F | GACATGTTCAGCTTTGTGGACCTC |
| Collagen 1a-R | GGGACCCTTAGGCCATTGTGTA |
| OCN-F | GACAAGTCCCACACAGCAACT |
| OCN-R | GGACATGAAGGCTTTGTCAGA |

**Supplementary Figure 1**

**
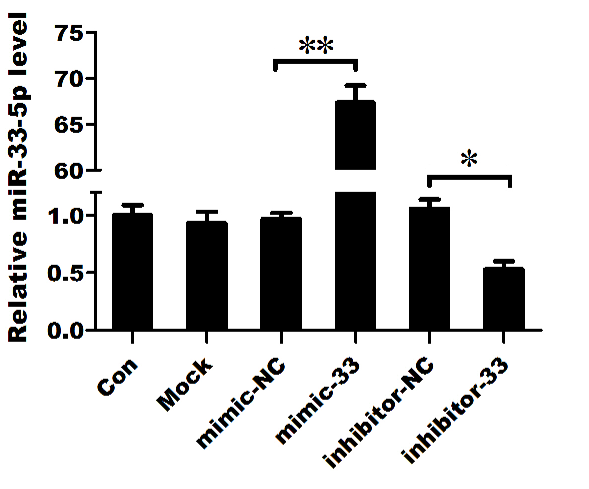
**

**Supplementary Figure 1. The regulative effects of miR-33-5p mimic and inhibitor on miR-33-5p level.** qRT-PCR analysis of changes in the expression of miR-33-5p in MC3T3-E1 cells after treatment with mimic-33, inhibitor-33 or their negative controls (100 nM) for 48 h. The data are expressed as the mean ± SD of three replicates each. **P* < 0.05, ***P* < 0.01 vs. the control.

**Supplementary Figure 2**

**
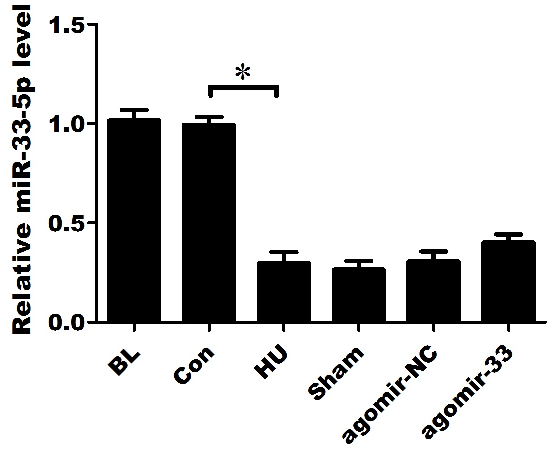
**

**Supplementary Figure 2. The miR-33-5p levels of each groups after the hindlimb unloading.** qRT-PCR analysis of the miR-33-5p expression in each groups after the 21 days of hindlimb unloading. The data are expressed as the mean ± SD of six replicates each. **P* < 0.05 vs. the control.
